# A Deep Learning Approach for Maximum Activity Links in D2D Communications

**DOI:** 10.3390/s19132941

**Published:** 2019-07-03

**Authors:** Bocheng Yu, Xingjun Zhang, Francesco Palmieri, Erwan Creignou, Ilsun You

**Affiliations:** 1 School of Computer Science and Technology, Xi’an Jiaotong University, Xi’an 710049, China; 2 Department of Computer Science, University of Salerno, 84084 Fisciano (SA), Italy; 3 Department of Information Security Engineering, Soonchunhyang University, Asan-si 31538, Korea

**Keywords:** D2D communications, deep learning, link activation, wireless networks, integer programming

## Abstract

Mobile cellular communications are experiencing an exponential growth in traffic load on Long Term Evolution (LTE) eNode B (eNB) components. Such load can be significantly contained by directly sharing content among nearby users through device-to-device (D2D) communications, so that repeated downloads of the same data can be avoided as much as possible. Accordingly, for the purpose of improving the efficiency of content sharing and decreasing the load on the eNB, it is important to maximize the number of simultaneous D2D transmissions. Specially, maximizing the number of D2D links can not only improve spectrum and energy efficiency but can also reduce transmission delay. However, enabling maximum D2D links in a cellular network poses two major challenges. First, the interference between the D2D and cellular communications could critically affect their performance. Second, the minimum quality of service (QoS) requirement of cellular and D2D communication must be guaranteed. Therefore, a selection of active links is critical to gain the maximum number of D2D links. This can be formulated as a classical integer linear programming problem (link scheduling) that is known to be NP-hard. This paper proposes to obtain a set of network features via deep learning for solving this challenging problem. The idea is to optimize the D2D link schedule problem with a deep neural network (DNN). This makes a significant time reduction for delay-sensitive operations, since the computational overhead is mainly spent in the training process of the model. The simulation performed on a randomly generated link schedule problem showed that our algorithm is capable of finding satisfactory D2D link scheduling solutions by reducing computation time up to 90% without significantly affecting their accuracy.

## 1. Introduction

In the last decade, the diffusion of smartphones, tablets, and other smart devices has led to an astonishing demand for ubiquitous mobile network access, and the rise of online services, such as video streaming, social network applications, and mobile gaming, has significantly increased the traffic load characterizing wireless communications. Typically, the same popular data and content may be requested multiple times at the same location by different users/devices, which results in an unnecessary waste of backhaul capacity and spectrum resources. In fact, the main cause of these problems is due to video on demand contents accessed in an asynchronous way (unlike live streaming and digital TV), so that the demands concentrate on a small set of popular contents that are requested by a significant number of devices, often located in the same cellular coverage area [1]. To solve these problems, it is possible to reconsider the current network architecture and explore more advanced communication models and paradigms. D2D direct communication is considered one of the most promising technological issues in next generation cellular networks [2]. 

It consists of establishing a direct communication channel between two nearby mobile users, sharing a common short-range radio coverage space, without traversing a base station (BS) or core network equipment [3]. It results in a very flexible communication model with unique advantages. First, due to its underlying short-range direct communication technology, D2D user equipment (UE) allows for higher transfer rates together with very limited end-to-end delays. Second, any direct proximity-based transmission that does not traverse centralized channel collection points (and potential bottlenecks) in the cellular infrastructure, such as the evolved Node B (eNB) in LTE networks, may be more resource-efficient than conventional cellular communication by saving energy and channel capacity, as well as improving spectrum utilization. Third, data are transferred locally, which is helpful for offloading cellular traffic and alleviating backhaul crowding [4]. To enhance the efficiency of content sharing and reduce the load on the eNB, it is important to maximize the number of simultaneous D2D transmissions, each associated to an active communication channel or link, within a specific coverage area. In particular, maximizing the potential number of D2D links within a cellular coverage area not only improves the spectrum and energy efficiency but also reduces transmission delay. However, such a maximization process poses three major challenges. First, interference between cellular and D2D users could critically affect their performance. Second, the minimum quality of service (QoS) requirements must be simultaneously guaranteed for both cellular and D2D communications. Therefore, the selection of suitable active links in each time interval (or slot, in presence of a slotted time evolution model) is fundamental to achieve the maximum number of simultaneous D2D links. Third, a highly flexible and scalable network infrastructure needs to support a large number of heterogeneous users as well as the deployment and interworking of a multiplicity of combined technologies, such as the ones characterizing the Internet of Things (IoT) and vehicular ad-hoc communications [5,6,7], so that the degree of complexity characterizing such infrastructure, both in terms of variables and degrees of freedom in the associated model, makes the traditional approaches provided by optimization theory more challenging than ever [3]. The research of scheduling for network resources also causes great concern about computational efficiency [8,9].

The D2D links scheduling problem, aiming at maximizing the number of active D2D links, consists of determining which D2D pairs can communicate simultaneously (by using direct channels) while guaranteeing that the remaining communications between cellular users are not affected. Since D2D users share spectrum resources with cellular users, interference is one of the major challenges in D2D link scheduling. Interference may be not only experienced between D2D users operating within the same area but, D2D transmission may cause cross-layer interference phenomena also involving cellular users. The interference occurring in D2D communication can be modeled by using both a graph-based model and a physics-based one. The maximum D2D links problem has been proven to be NP-hard in both models [10]. Most research is based on the graph model (e.g., [11,12,13,14,15], but such model is too simplistic to simulate how the signal strength attenuation is affected by distance for D2D users which share the same cellular spectrum resources. On the contrary, the physics-based model adopted in this paper reflects the physical reality in a more precise and realistic way.

In real production networks, scheduling decisions must be taken online and in real-time so that the available optimization strategies aiming at solving offline the NP-hard linear programming-based problem formulations are not acceptable at all. In order to satisfy the strict delay demands of online operations, the complexity of a link scheduling algorithm must be significantly reduced, eventually applying some heuristic strategy leading to sub-optimal but yet acceptable results. However, this may affect network performance, depending on the achieved distance from the optimum solution. Therefore, developing efficient and effective heuristic-based strategies to solve the link scheduling problem becomes a really challenging task when implementing D2D communication in cellular network scenarios. In recent years, machine learning has emerged as a successful approach for coping with complex problems and has been widely applied to many fields, such as hyperspectral image classification, ship detection, and wireless communications [16,17,18,19]. With the success of massive parallel computing environments, also empowered by the use of graphics processing unit (GPU)-based acceleration frameworks, machine learning has emerged as a successful approach for coping with complex problems in the wireless communications area. In particular, deep learning, a promising subset of machine learning, has been applied to wireless networks optimization and big data processing. The purpose of deep learning is to simulate complex functions through a predefined model consisting of neuron units. In the deep learning training process, appropriate weight values are determined through calculation and tuning between neurons, a process which has the goal of extracting knowledge/information, in form of specific features from input data. Then the trained-model is able to make accurate classification or prediction decisions based on such features [20]. Unlike traditional machine learning methods that heavily rely on relevant domain experts to extract features from data, the deep learning model automatically acquires a sample feature through multiple hidden layers consisting of neuron units to achieve classification or prediction [21]. 

In this paper, we leverage the physical interference model and develop a novel machine learning-based approach for achieving the maximum number of D2D links within a cellular coverage area. We use deep neural network (DNN)-derived features from D2D link information to make time-efficient and near-optimal decisions in the operation phase through a real-time optimization strategy driven by the neural network itself. The DNN model is trained by pre-computed samples to find out the complex relationship between link information and D2D link activation. In doing this, we consider a more realistic and accurate signal-to-interference-plus-noise ratio (SINR) interference model that considers both cellular users and D2D users. We also accomplish extensive simulations to assess the performance of the DNN-based method and highlight its effectiveness. The trained-DNN approach can generate approximate solutions quite near the optimum by immediately achieving a satisfactory quality of the outputs. The numerical of simulation shows the superiority of the DNN-driven solution in terms of the time-cost of the operations involved respect to iterative optimization algorithms.

Our main contribution can be summarized as follows:

(1) In this paper, we propose a cutting-edge learning-based method for solving the maximum D2D links problem. Using the training samples generated by a conventional method, we train a deep learning model to simulate the complex relationship between link information and link activity. Due to computational burden transferred to the training phase, the algorithm significantly reduces the computational overhead.
(2) We introduced a sizable D2D communication network under an SINR interference model. Furthermore, we present the value of a two-layer interference — a D2D layer and cellular layer—and then formulate a maximum active D2D links problem subject to SINR constraints.
(3) We adjust the parameters of the learning model depending on simulation results to get an optimal output. The experiments show that our algorithm can reduce more than 90% the time-spent.
(4) Through simulation results, we analyze how some factors, such as SINR and the coverage of the BS, affect the number of active D2D links. Based on such analysis, we can efficiently schedule D2D links under different wireless network environments.

## 2. Related Work

Link scheduling plays a crucial role in meeting strict QoS requirement in wireless networks. Maximum link scheduling is an important sub-problem of link scheduling. The authors of [10] proved for the first time that link scheduling under a SINR model is NP-hard and proposed an algorithm for the one-shot link scheduling problem. The algorithm first separates the problem instance into disjoint link classes and then schedules each link class by using a greedy method. The work in [22] firstly explored a one-slot algorithm for maximizing the number of links with a constant approximation guarantee, and then an extended algorithm with O(logn) (*n* is total number of links) computational complexity was introduced for a minimized-length schedule. However, none of the above papers considered the ambient noise. A method based on partitioning was presented in [23] to find the independent link set with the maximum weight. In [24], a distributed greedy algorithm for maximizing the number of links, subject to interference constraints was proposed. In these approaches, the improvements achieved in terms of optimization quality could result in a hugely higher computational complexity and, hence, an increment in overall runtime performance. A low-complexity scheduling scheme named DistGreedy based on a link-conflict graph has been proposed in [25]. The algorithm repeatedly removed the active links and blocked links in contention slots until the graph was left empty. The authors of [26] attempted to find a maximum-weighted subset of communications without spectral splitting at the individual time unit. In this model, *A* denotes a set of communication tasks, and each request, a∈A, has a demand d(a)∈(0,1]. If d(a)∈(1/2,1], it is a heavy request, otherwise it is a light request. The algorithm considered both heavy requests and light requests of *A* for determining the link schedule. In [27], a maximum tolerance and minimum (MTMA) model based on a greedy schedule was studied for maximum link scheduling in one-slot. The experiments showed that the algorithm improved 28–64% of the current algorithm. The authors in [28] developed a distributed greedy heuristic for a *k*-hop link schedule. Because of interference from two layers, the algorithm for the D2D links schedule was much more complicated than above schemes.

With the development of the IoT, due the increased complexity of the network structure, link scheduling experiences new difficulties in meeting QoS requirements when using a traditional optimization method. In recent years, deep learning, has been promisingly applied to wireless networks optimization and big data processing [21,29,30]. In [31], the authors considered solving a resource allocation problem for D2D communication using a Q-learning-based method. In [32], the authors applied deep learning to reduce the complexity of solving a wireless networks optimization problem. The authors in [33] proposed a resource allocation strategy using cooperative reinforcement learning. The target of their algorithm was to maximize the throughput of the system by selecting the proper level of power for the resource blocks of the cellular user and D2D pairs. In [34], the authors adopted deep learning to solve the objective function of maximizing the weighed sum rate over *N* D2D users and demonstrated that link scheduling does not necessarily require the exact channel estimates. In [35], the author adopted a method based on reinforcement learning to solve the resource scheduling for vehicle-to-vehicle (V2V) communications based on D2D. In this article, each vehicle, regarded as an agent, made decisions by itself. Since they do not require global information, decentralized scheduling methods are characterized by a low overhead. Experiments have shown that the proposed algorithm can effectively schedule limited links with minimized interference under delay constraints. Solving the maximum active D2D links problem under interference constraints by deep learning is promising.

## 3. System Model

**Figure 1:**
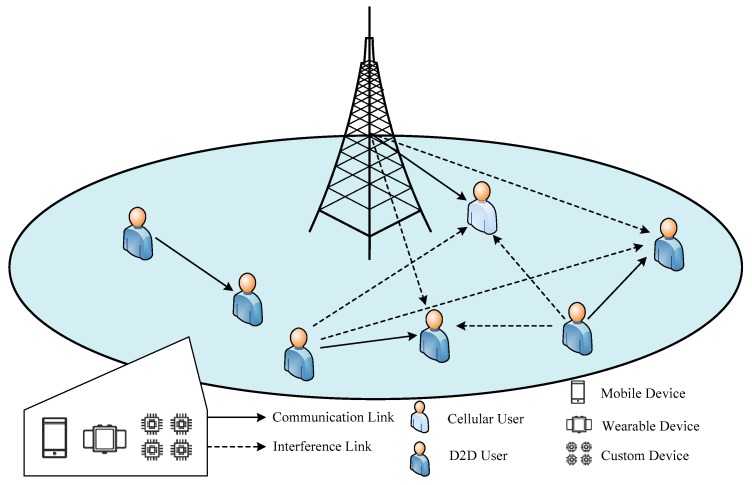
System model.

We considered that D2D communications occur within a single cell of a cellular system and share the system’s downlink resources. In this scenario, a cellular device may suffer interference phenomena introduced by D2D communication activities. D2D transmitters can also interfere with the eNB. Due to the strong interference management capability of the eNB, it is necessary to schedule links in order to allow downlink connectivity for cellular device communications. In the proposed system model, D2D devices are randomly distributed under the coverage of the same eNB. As shown in Figure 1, the heterogeneous network consists of a single eNB for serving cellular devices and *M* D2D-capable devices. Let $V=\left\{V_{1},V_{2},\cdots ,V_{M}\right\}$ denote the set of D2D users in the network. To make full use of available spectrum resources, we allowed multiple D2D devices to simultaneously use the same downlink channel of cellular devices. Two D2D devices (a D2D pair) can establish no more than a direct communication. According to the SINR model, the SINR involving a single D2D sender–receiver pair (*i,j*)(i,j∈V) in the presence of a cellular device *C* connected to an eNB operating in the same cell is:

(1) $$SINR_{D}=\frac{P_{i}g_{ij}}{P_{B}g_{C}+\eta }$$

where $P_{i}$ is the transmit power characterizing the D2D sender *i*, *g_ij_* denotes the propagation attenuation (link gain) modeled as *g_ij_* = *d_ij_*^−α^ (*d_ij_* denotes the distance between D2D pair and α ≥ 1 is a constant path-loss exponent), $P_{B}$ is the transmission power of the eNB, $g_{C}$ is the gain of a cellular device *C* connected to the eNB concurrent to *i*, and η is the ambient noise. Clearly, the signal power transmitted by the eNB to *C* is perceived at *i* as interference. Let $\gamma_{C}$ and $\gamma_{D}$ denote the minimum SINR threshold of the cellular device and D2D device, respectively. If the condition $SINR_{D}\geq \gamma_{D}$ is held, the D2D receiver *j* successfully receives a message from D2D sender *i* (the D2D pair of devices communicate successfully). We denote the set of *N* pairs *A* that are able to successfully perform D2D communication as $A=\left\{A_{1},A_{2},\cdots ,A_{N}\right\}$. The notation used in this paper is shown in Table 1.

There are four conditions in the model: (1) Any D2D terminal device can communicate with another within the same cell; (2) a node can be a sender or receiver, but a node can send to at most one receiver or receive from at most one sender; (3) the D2D receiving user can estimate the link state information (channel state information, CSI) according to the received signal; and (4) the eNB controls channel resource allocation in a centralized way.

## 4. Problem Formulation

As mentioned above, multiple D2D communications are allowed within the same cell by sharing spectrum resources with cellular users. Therefore, the inter-layer interference between devices involved in cellular communication through the eNB and devices performing D2D data transfers as well as intra-layer interferences between the different D2D pairs can be experienced and must be correctly managed. When the number of D2D communication links heavily increases, the interference caused by the sharing of the same spectrum resources will affect the reliability of cellular device communications and even reduce the number of potential D2D links. In general, a device requiring spectrum resources for cellular communication has a higher priority than a device competing for establishing a D2D communication channel. Therefore, the resource scheduling problem is related to maximizing the amount of D2D links while ensuring the QoS of the cellular device, which selects a subset of *A* with the biggest cardinality under $SINR_{D}\geq \gamma_{D}$. Based on the above analysis, we started from a conventional link scheduling scheme which guaranteed the communication reliability of the cellular users and maximized the active D2D links. Due to the NP-hard nature of the underlying problem, we used a DNN to optimize the above scheduling algorithm and reduce its running-time.

In order to properly formulate the problem, at first, we need to analyze the interference in the cellular system (i.e., within the cell of inters). Here, when the pair of devices involved in direct D2D communication reuses the downlink spectrum, the signal received by a generic cellular device *C* consists of the expected signal from the eNB, with the addition of the interference from the D2D layer and the ambient noise. In detail, by using the physical model, the SINR of the generic cellular device *C* is defined as follows:

(2) $$SINR_{C}=\frac{P_{B}g_{C}}{\sum_{\left(i,j\right)\in A}x_{ij}P_{i}g_{iC}+\eta }$$

where $P_{B}$ is the transmission power of the eNB, $P_{i}$ is the transmission power of the D2D device *i*, $g_{iC}$ denotes the link gain from D2D sender *i* to cellular device *C*, $g_{C}$ is the gain of the cellular device connected to the eNB, and η is ambient noise. To ensure the performance of the cellular device, $SINR_{C}\geq \gamma_{C}$ should be satisfied. Analogously, in the D2D layer, the signal perceived at the D2D receiver *j* side consists of the one transmitted by *i* affected by the interference from both the cellular and D2D layers. As such, the SINR at the D2D device level is:

(3) $$SINR_{D}=\frac{P_{i}g_{ij}}{\sum_{\left(k,j\right)\in A,\;k\neq i}x_{kj}P_{k}g_{kj}+P_{B}g_{Bj}+\eta }$$

where $g_{ij}$ is the channel gain from the D2D sender *i* to receiver *j* and $g_{Bj}$ is the channel gain from the eNB to D2D device *j*. When $SINR_{D}\geq \gamma_{D}$, the D2D links perform normally.

To maximize the total amount of admissible D2D pairs, we need to consider the following maximization link utility problem, subject to interference constraints associated to the performance of D2D devices and the cellular user:

(4) $$[MaxL]\;L^{*}=max\sum_{\left(i,j\in A\right)}x_{ij}$$

(5) $$s.t.\;\sum_{j\in V:\left(i,j\right)\in A}x_{ij}+\sum_{j\in V:\left(i,j\right)\in A}x_{ji}\leq 1,\;i\in V$$

(6) $$\underset{j\in V:\left(i,j\right)\in A}{\sum }x_{ij}=y_{i}$$

(7) $$P_{B}g_{c}\geq \gamma_{C}\left(\underset{i\in V}{\sum }P_{i}g_{ic}y_{i}+\eta \right)$$

(8) $$P_{i}g_{ij}x_{ij}+M_{ij}\left(1-x_{ij}\right)\geq \gamma_{D}\left(\underset{k\neq i}{\sum }P_{k}g_{kj}y_{k}+P_{B}g_{Bj}+\eta \right)$$

(9) $$x_{ij}\in \left\{0,1\right\},\;\left(i,j\right)\in A$$

(10) $$y_{i}\in \left\{0,1\right\},\;i\in V$$

The objective (4) maximizes the number of active D2D links. The binary variables *x_ij_* and *y_i_* are respectively associated to the presence of an active D2D pair from the devices *i* to *j* and to the capability of D2D sender *i* to perform D2D communication. The constraints (5) and (6) state that a D2D device can send to at most one receiver or receive from at most one sender; they also state that each device terminating a link must be D2D-capable, whereas the constraints (7) and (8) formulate, respectively, the SINR requirement of the cellular device and D2D devices. The use of a binary variable to control whether a linear constraint is active is a very well-known modeling trick in integer linear programming [36]. The solution of the problem [*MaxL*] can be obtained by using an exact algorithm or a heuristic one. The exact algorithm is typically based on the use of the branch and bound [37] and dynamic programming methods [38]. Though such methods can obtain an optimal solution, their computational complexity is large and only suitable for small scale problems. On the other hand, the heuristic algorithm is not based on finding the optimal solution of the problem, but it expects to obtain a near global optimal solution in an acceptable time. If $x_{ij}=1$, (8) constrains the SINR to be at least $\gamma_{D}$ for $x_{ij}=0$. When no D2D links are active for the pair (*i, j*), the constraint is always satisfied for a sufficiently large $M_{ij}$. Large values for $M_{ij}$ can easily lead to numerical problems and to weak linear relaxations ([39,40]). The choice of such big numbers $\mathrm{for}\;M_{ij}$, known as Big-M in integer programming, is used to turn on or off some inequality constraints when necessary and can potentially result in difficulties when trying to solve an integer linear programming problem like *MaxL* that relies heavily on the bounds of continuous relaxation to be solved in a computationally acceptable time. That is, an improper selection of the *M_ij_* values can potentially result in a very weak continuous relaxation. Moreover, the gain values in (8) may vary significantly in magnitude and, hence, may introduce other numerical difficulties in solving the problem.

## 5. Deep Learning-Based Link Scheduling

In real networks, users’ positions, channel conditions, and data requests vary frequently, so any effective scheduling algorithm must be able to make decisions in real time. In addition, the potential number of D2D links increases exponentially with the number of D2D devices *M* operating within the same cell. In scenarios characterized by large *M* values, selecting all the feasible D2D links in order to offload cellular communications becomes extremely time consuming. To develop a time-efficient algorithm, we designed a machine learning-based solution aimed at supporting and simplifying the resolution of the aforementioned *MaxL* problem, based on properly training a DNN. 

### 5.1. General Deep Learning

**Figure 2:**
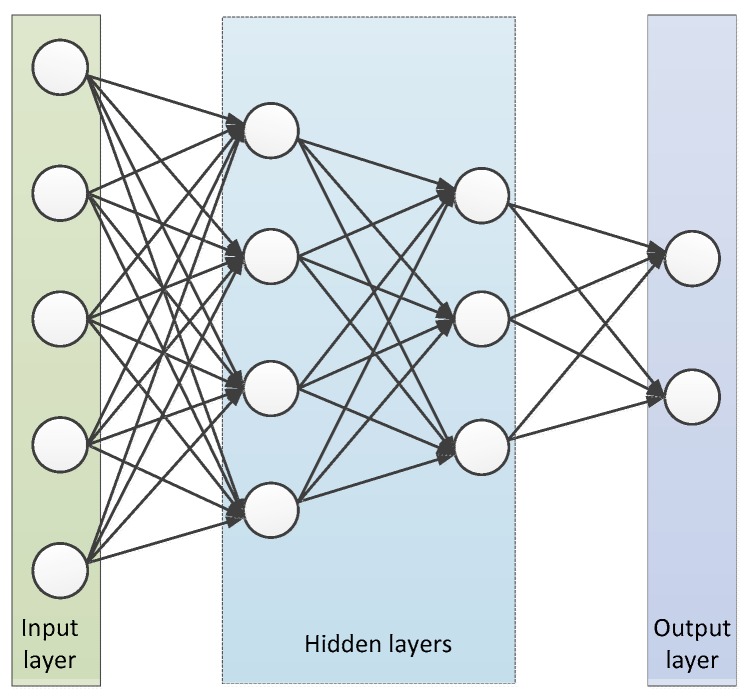
Learning structure.

Figure 2 is the general architecture of the deep learning-based solution, in which the input layer represents the features of the D2D pairs, and the output layer reports the D2D link activation result. In the most general case, the parameters of hidden layers which consist of weights and the values of neurons are used to connect the input layer and the output layer. The parameters of each hidden layer are determined by the previous layer. In the process of training a typical learning model, we first collected labeled input data to be used as a training set. Then the training set was fed to input layers during the training phase by acquiring the results from the output layer, which may differ from the expected values. The difference between the output value and the expected value can be calculated by a loss function. Weights can be modified by a backpropagation (BP) method in order to minimize the above difference or loss function. The training process of the learning network is meant to adjust the weights through the training samples. When entering a test data set, the learning framework will generate a vector, which is an estimate of labeled value. The performance of a trained learning model depends on the differences between the output vector and real value. Due to the fact that the performance of a deep learning model is greatly influenced by the model parameters, it is difficult to train a deep learning network [41]. For instance, in presence of a limited data set, if the training model is designed in a way that is too complicated and tries to approximate a complex data relationship by using a noise sample, it results into overfitting [42]. Conversely, if the design of the model is too simple to fully simulate data correlation, the model can lead to underfitting. Both of these issues affect the generalization ability of the learning model. When the deep learning model faces large-scale data, both a high computational complexity and a long time-spending are introduced by the sum of the loss functions using all the data as the training objects, so it is important to select an appropriate batch size for training. In this paper, a series of parameters were compared through experiments to optimize the performance of the learning model.

### 5.2. Deep Belief Network

**Figure 3:**
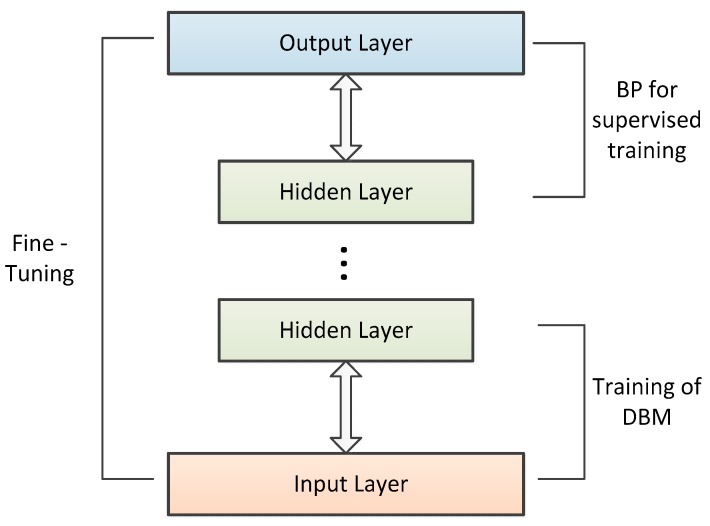
Training process of deep belief network.

A deep learning framework is applied in the lower-levels of unsupervised learning networks [43]. We adopted the deep belief network (DBN) method to solve our link scheduling problem [44]. A DBN is a neural network composed of multiple layers of restricted Boltzmann stack machines (RBMs). An RBM consists of two kinds of layers. One is the visible layer for inputting samples. The other one, named the hidden layer, is used for extracting features. In an RBM, there is no connection in the same layer, and each visible layer is connected to a hidden layer via symmetric weights. Since the weights are all symmetric, an RBM can not only infer the state of neurons using hidden layers, it can also use them to reconstruct the input values. Since an RBM is not sufficient to extract complex information from the input, a DBN was adopted in the paper. A DBN consists of a visible layer as a bottom layer and other hidden layers, and the training process is mainly divided into two steps, as shown in Figure 3. Firstly, the layer-wise training strategy is adopted for an RBM. The input vector is used to train the hidden layer, and the output of the hidden layer is regarded as the input data vector for the higher layer. Secondly, the output layer of the DBN sets up the BP network, which receives the output vector from an RBM as its input vector and trains the classifier under supervised learning. Each layer can only guarantee the optimal output feature vector by adjusting weights, but it cannot make sure that the final output of a DBN is the optimal value. The DBN can use the difference between the output value and labeled value from the backpropagation network for the fine-tuning of the whole network. The training process of a DBN network can be used as the initialization of the weight parameter of a deep BP network, which makes the DBN overcome the shortcomings of the random initialization weight parameters that makes the BP network easily fall into the low optimum values and spend a long time training.

### 5.3. DNN-Based Approach

#### 5.3.1. Design of Input layer

The general model creates D2D link activation with information from the input layer. Information from the input layer should be able to indicate a variety of valuable information, including the location of D2D pairs and cellular devices. To this end, we define a |(*M* + 1 )| × |( *M* + 1 )| matrix *N* for D2D users and cellular users, whose entries are specified as:

(11) $$N_{ij}=\{\begin{array}{c} d_{ij}\;if\left(i,j\right)\;are\;an\;active\;D2D\;pair\\ 0\;otherwise\; \end{array}$$

The input matrix first needs to be transformed into a ${\left|\left(M+1\right)\right|}^{2}$ vector to be used as an input to the learning framework. Because the DBN needs to be consistent with the sigmoid function, the node can only accept values in the range [0,1]. We need to normalize the input vector by dividing the largest element among the input vectors. We chose a maximum communicable distance of D2D pair as $d_{max}$ to normalize the vector in order to avoid the influence of normalization on the learning result.

In fact, $d_{max}$ is calculated by the SINR model in network optimization problems in order to get feasible solutions due to limitation of the capabilities of the network. Summarily, the standardized D2D information vector can be fed to the learning model, which is:

(12) $$\overset{ˇ}{d}=\frac{d}{d_{max}}$$

#### 5.3.2. Design of Output layer

The learning model is designed to predict whether each D2D link is active in order to maximize the amount of D2D communication links. In a classification model, the output vector consists of categories of labeled input values. If *N* data need to be classified, the corresponding output should be an *N*-dimensional vector. Therefore, the length of the output vector should be the same as the total number of D2D pairs. In our model, the number of elementselement in the output vector is *M.* The value of the output is transposed in the rangetransformed between 0 and 1 by softmax to represent the probability of D2D link activation. The maximum probability value is set to 1, where element 1 indicates that the D2D pair is active. The evaluation of the output vector is used to tune the learning model weights by using the BP method.

#### 5.3.3. Training Set

Once the input and output layers are settled, the training set consisting of pairs of D2D location information and a D2D links activation state must be constructed. The training set is usually obtained from historical data or an off-line solution to sample problems. In this paper, we randomly generated D2D user location information and simulated the transmission state of the channel. A conventional optimization algorithm was exploited to solve the D2D link schedule problem and produce labeled training samples containing link information and a binary link state value. We present the training process in Algorithm 1.


**Algorithm 1: Procession of Training Set Generation**

Generate a random location set of D2D nodes and cellular node;Formulate the D2D link schedule problem;initialization: *n = 0*;**while** not at end of this document **do**  Solve the optimization problem by conventional method;  Add labeled solution to the training set;  *i = i + 1*;
**end**



#### 5.3.4. Training Process

Algorithm 2 summarizes the training process of the learning framework. First, Algorithm 1 generates the training set. Then, the DBN is unsupervised and trained by a multi-layer RBM. In the pre-training phase, real network data with initial random weights are used to train the DBN. The BP method is applied to the supervised training. After multiple rounds of training, the weights in the DBN are fixed. Finally, the BP method is performed on all layers of fine-tuning.


**Algorithm 2: Training Process of a DBN**

**Input:** Training Set;The number of layers: *L*, weight of *lth* layer: $w^{\left(l\right)}$;**for**l=1⋯L do  initialization: $w^{\left(l\right)}\to 0$;   extract feature $h^{\left(l-1\right)}$;  train $w^{\left(l\right)}$ of RBM of *lth* layer using $h^{\left(l-1\right)}$;
**end**
use BP method to adjust the weight;fine-tuning the parameters of the whole layer**output:** the state of D2D links


## 6. Performance Evaluation

**Figure 4:**
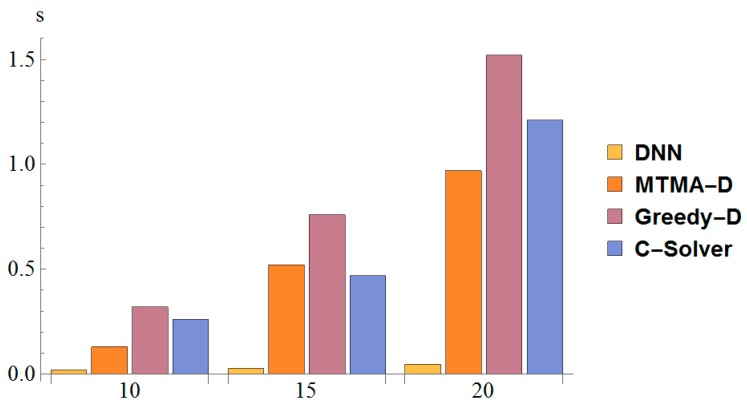
Comparison of computation time.

In our proof-of-concept evaluation scenario the nodes are randomly placed on an area of 250 m^2^. The channel gain is $g_{ij}=d_{ij}^{-3}$, where $d_{ij}^{}$ is the distance between D2D users *i* and *j*. The detailed simulation parameters are given in Table 2:

In Figure 4, we compare the time required (seconds) for reaching optimality with the DNN-based algorithm against the ones experienced by using respectively the conventional integer linear programming optimization scheme solved with CPLEX (C-Solver) and the maximum weighted links scheduling (MWLS) algorithm, a greedy approach for D2D link scheduling under the SINR constraints based on [27,28]. The TensorFlow framework has been used to train the DNN-based algorithm. We determined the average calculation time for each case. From Figure 4, we can see that the computation time based on the DNN algorithm was considerably reduced when compared to the ones characterizing the other optimization approaches. In addition, as the scale of the instance increases, the computational time of the other algorithms increases as well according to a huge growth trend. Instead, for the DNN-based approach the computational time growth with the problem scale is not so obvious.

In Table 3, we compare the effect of the number of hidden layers in our solution. To keep the comparison fairer, both of the models shared the same number of hidden units in total. The two-layer-deep model had 60 units per layer, while the model with three layers had 40 units per layer. We focused on time and accuracy as comparison metrics. In terms of time, we observed the training time and testing/validation time of the learning model with two and three hidden layers. To estimate accuracy, we relied on a binary variable denoting the link status: 1 meant that the D2D link was activated and the other link was asleep. The binary accuracy of the output link status has been used to show the performance of the model in training and testing. Table 3 shows that the three-layer structure spent more time in the training and testing than the two-layer model. Since the total number of neurons was the same, the accuracy only experiences little differences.

**Figure 5:**
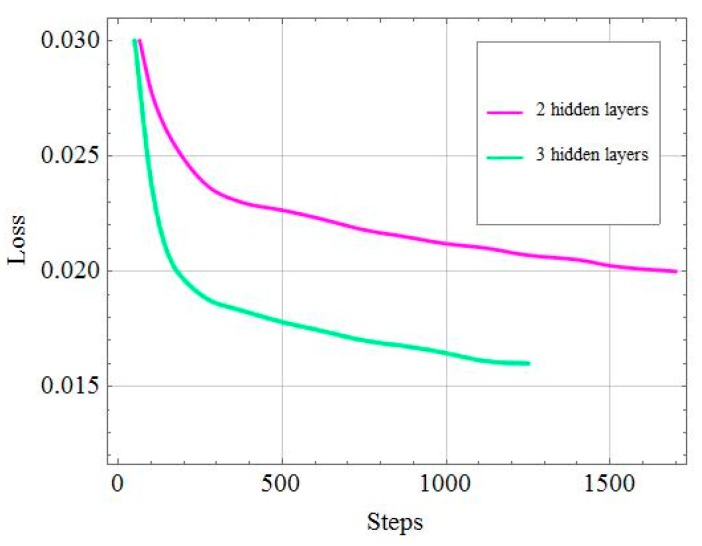
Loss over time on the training set for different depths of the deep neural network (DNN).

In Figure 5, we set 60 units per layers for both models. According to Figure 4, it is reasonable to keep the depth of the DNN limited to three hidden layers. Training accuracy is better for a three-layer network while keeping the testing time within acceptability bounds. The difference in terms of accuracy between the two models can be explained by the number of weights that need to be trained. Weights are several times more numerous in the three-layer than in the two-layer model. Due to the vanishing gradient, we can observe the training loss declined gradually as the number of training steps increase.

**Figure 6:**
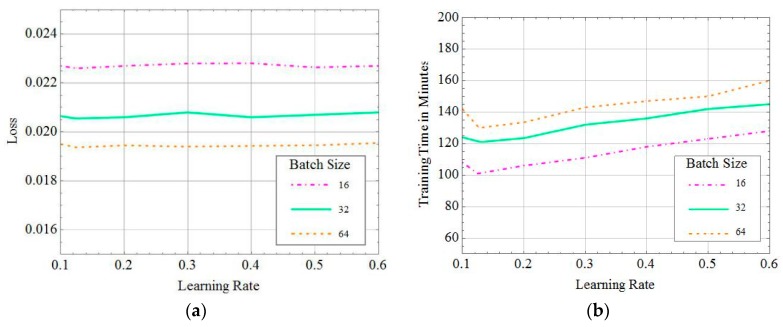
Effect of the learning rate and batch size on the training phase. (**a**) Effect of the learning rate and batch size on training loss. (**b**) Effect of the learning rate and batch size on training time.

It is critical to choose a proper batch size to effectively prevent the model from underfitting and overfitting. We compared the validation loss and training time with different batch sizes and learning rates, and the results are shown in Figure 6. The values of learning rate and batch size were decided by an experimental comparison with a constant value of epoch. From Figure 6a, we can see that the validation loss became smaller as the batch size increased. This is because the learning model approximated better with the characteristics of the training set with a bigger data size. Meanwhile, validation loss was impacted limitedly under different learning rates. As it can be seen from Figure 6b, the training time decreased with an increase of the batch size. Moreover, when the learning rate increased, the training time first dropped and then rose. This is because the rate of gradient descent increased with the learning rate. However, an excessive learning rate can lead to an excessive parameter update and to an increase of training time. 

As a result, the learning rate was equal to about 0.12 with the least training time. Then, we investigated the details of how variations of batch size affect training time, as shown in Table 4. Table 4 indicates the trade-off between validation loss and training time at the batch size of 64.

**Figure 7:**
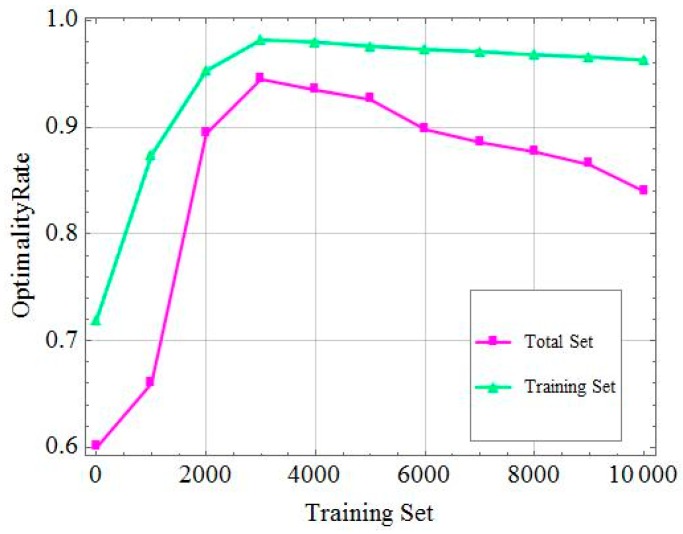
Optimality rate of different training set sizes.

Figure 7 shows the accuracy of the results of the DNN-based approach varying with different dataset sizes. We used the optimality rate as a metric to check the probability that the result was the label value. For example, when the optimality rate was equal to 0.8, the output generated by the DNN had an 80% probability of being the label value. We tested the accuracy of the DNN algorithm by using only the training set and the entire data set. According to Figure 7, the optimality rate increased with the size of the training set. When the test set was approximately 3000 instances, the output got the best optimization result of 95% or more. At the same time, the average accuracy of the output value based on the DNN algorithm can reach more than 90% of the results of a conventional optimization algorithm. We can observe that the optimality rate did not change much as the training set increased, but it gradually decreased when the total data set has been used. This is because the noise present within data got larger as the data set size increased, and during the training process, the model can learn some relationships by such noisy data. The learned model can perform well for a training set, but it cannot achieve the same accurate outputs when using all the data. In our simulation, the training set size of 3000 was the optimal choice.

**Figure 8:**
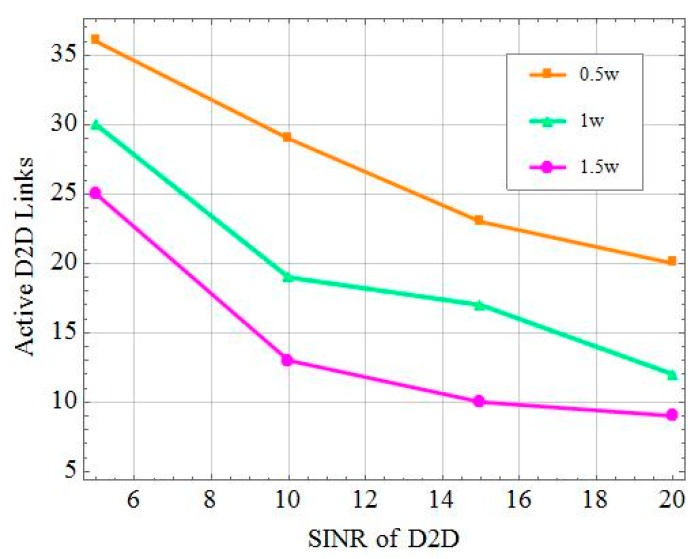
The impact of activity on device-to-device (D2D) links under different SINRs and different base station powers.

Figure 8 compares the impact of the SINR threshold of the D2D receiving user on the number of D2D links that the system can activate at different base station transmit powers. As seen in Figure 8, the more D2D pairs that can be activated by the system, the smaller is the transmit power of the base station. Conversely, the increase in transmit power of the base station resulted in a reduction of the number of active D2D links. This is because a greater transmit power of the base station implied more interference for the D2D users, resulting in fewer admissible D2D pairs and more D2D links that could be allowed to simultaneously communicate with a smaller SINR threshold. Otherwise, the amount of active D2D pairs decreased in the cell with a larger SINR. Since more D2D devices can receive data from others with lower SINR thresholds, the amount of D2D activity decreased with the strength of the interference with the base station and the peer D2D users.

**Figure 9:**
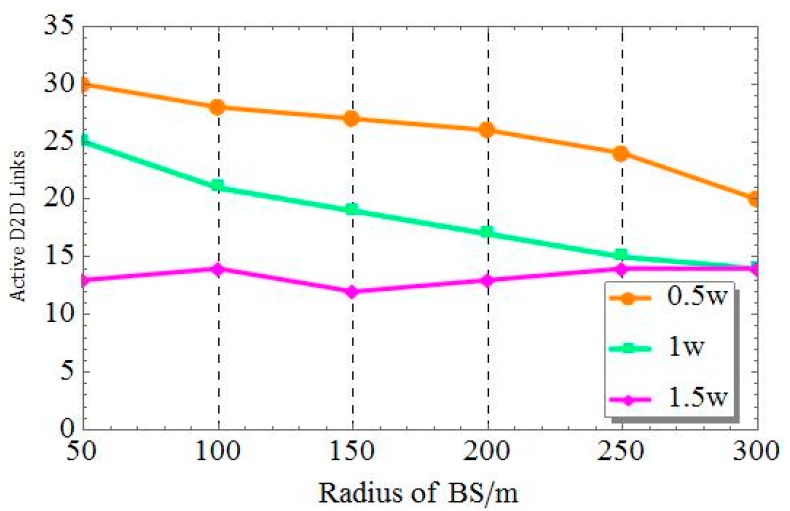
The number of active D2D links under different radii and different base station powers.

In Figure 9, we show the change in the number of D2D links with respect to the change of D2D distribution radiuses and base station transmit powers. It can be seen from Figure 9 that when the base station transmit powers are 0.5w and 1w, the D2Ds device distribution range increases and the number of D2D links decreases slowly because the number of potential devices involved in D2D communication increases with the growth of the distribution radius, thus causing severe interference. In this case, the transmission distance of D2D links increased, and the receiving signals became easily affected by other D2D devices and the eNB activity. When the transmit power of the eNB was 1.5w, the amount of D2D links declined slightly and then turned to grow slowly. The overall change was not significant because the distance between the D2D devices became larger as the distribution radius increased. During signal transmission, the amount of potential D2D links grows as the distribution radius increases. That is because D2D equipment can potentially be located farther from the eNB, and the interference from the eNB can be reduced. 

## 7. Conclusions

Creating maximum simultaneous D2D link schedules for dealing with increasing traffic loads is a challenging problem in modern cellular scenarios due to the need for reducing the load on base stations and because of their high computational complexity. To date, many link scheduling optimization algorithms have been developed, but most of them are not well suited for working online and hence cannot meet the real-time requirements of modern network infrastructures. In this paper, we used a machine learning-based strategy to maximize the number of simultaneously active D2D links without interfering with cellular communications. Based on the physical interference model, we designed a link scheduling algorithm based on a DNN to predict device activity in forming D2D links in order to reduce computation times-costs. Since the application of deep learning is still an emerging research topic in network scheduling, we empirically determined the proper operating parameters via specifically crafted experiments aiming at optimizing the model performance. The parameters chosen in this way were the number of hidden layers, the size of the training set, the batch size, and the learning rate. Simulation experiments showed that the proposed approach can reach more than 90% of the optimum results quality. At the same time, we learned that the influence of base station power is greater than the threshold of an SINR on the number of D2D links. Through the experiments, we also learned that the influence of the number of D2D links changes in presence of different base stations’ coverage areas. Furthermore, we also analyzed the reasons for these factors. For future work, we are going to consider more solutions, like transfer learning and reinforcement learning for the more general case that D2D links may be scheduled in a multi-cell.

## Figures and Tables

**Table 1:** Summary of notation.

Symbol	Meaning
*V*	Sets of D2Ds
*A*	Sets of D2D pairs
*C*	Cellular user
η	Ambient noise
α	Path-loss exponent
$P_{i}$	Transmit power of D2D *i*
$P_{B}$	Transmit power of the eNB
$P_{C}$	Transmit power of cellular user
$g_{ij}$	Channel gain between two D2D (*i* and *j*)
$g_{C}$	Channel gain between the eNB to cellular user
$d_{ij}$	Transmission distance of two D2D (i and *j*)
$d_{iC}$	Transmission distance between D2D i to cellular user

**Table 2:** Parameters setting of simulation.

Parameter	Value
Cell Radius/m	250
Number of Cellular Users	1
Transmit Power of D2D	0.01
$\gamma_{C}$	10
$\gamma_{D}$	10
η/mw	$10^{-10}$

**Table 3:** Results of the comparison between different number of layers.

Metrics	2 Hidden Layers	3 Hidden Layers
Training Time	1h 24min	1h36min
Testing time (on 10k)	0.247032s	0.233568s
Binary accuracy on training	0.99458	0.99356
Binary accuracy on testing	0.99320	0.99261

**Table 4:** Results of the comparison between different batch size.

Batch Size	Training Time	Training Loss
16	1 h 41 min	0.0226
32	2 h 01 min	0.02053
64	2 h 10 min	0.0194

## References

[1] GSMA The Mobile Economy 2018 (white paper). https://www.gsma.com/mobileeconomy/.

[2] Asadi A., Wang Q., Mancuso V. (2014). A survey on device-to-device communication in cellular networks. IEEE Commun. Surv. Tutorials.

[3] Lin X., Andrews J., Ghosh A., Ratasuk R. (2014). An overview of 3GPP device-to-device proximity services. IEEE Commun. Mag..

[4] Choudhary G., Kim J., Sharma V. (2018). Security of 5G-Mobile Backhaul Networks: A Survey. J. Wirel. Mob. Netw. Ubiquitous Comput. Dependable Appl..

[5] Gritti C., Önen M., Molva R., Susilo W., Plantard T. (2018). Device Identification and Personal Data Attestation in Networks. J. Wirel. Mob. Netw. Ubiquitous Comput. Dependable Appl..

[6] Arena F., Pau G., Collotta M. (2018). A survey on driverless vehicles: from their diffusion to security features. J. Internet Serv. Inf. Secur..

[7] Lei M., Zhang X., Ding H., Yu B. (2018). Fairness-Aware Resource Allocation in Multi-Hop Wireless Powered Communication Networks with User Cooperation. Sensors.

[8] Liu L., Cao X., Shen W., Cheng Y., Cai L. Dafee: A decomposed approach for energy efficient networking in multi-radio multi-channel wireless networks. Proceedings of the 35th Annual IEEE International Conference on Computer Communications.

[9] Mensah K.K., Chai R., Bilibashi D., Gao F. (2016). Energy efficiency based joint cell selection and power allocation scheme for HetNets. Digital Commun. Networks.

[10] Goussevskaia O., Oswald Y.A., Wattenhofer R. Complexity in geometric SINR. Proceedings of the 8th ACM International Symposium on Mobile Ad Hoc Networking and Computing.

[11] Moscibroda T., Wattenhofer R., Weber Y. Protocol Design Beyond Graph-Based Models. http://citeseerx.ist.psu.edu/viewdoc/download?doi=10.1.1.87.4443&rep=rep1&type=pdf#page=43.

[12] Maheshwari R., Jain S., Das S.R. A measurement study of interference modeling and scheduling in low-power wireless networks. Proceedings of the 6th ACM Conference on Embedded Network Sensor Systems.

[13] Li Q., Negi R. (2012). Maximal scheduling in wireless ad hoc networks with hypergraph interference models. IEEE Trans. Veh. Technol..

[14] Wang W., Wang Y., Li X.Y., Song W.Z., Frieder O. Efficient interference-aware TDMA link scheduling for static wireless networks. Proceedings of the 12th Annual International Conference on Mobile Computing and Networking.

[15] Wang C., Yu J., Yu D., Huang B., Yu S. (2016). An improved approximation algorithm for the shortest link scheduling in wireless networks under SINR and hypergraph models. J. Comb. Optim..

[16] Sze V., Chen Y.H., Yang T.J., Emer J.S. (2017). Efficient processing of deep neural networks: A tutorial and survey. Proc. IEEE.

[17] Peng J., Sun W., Ma L., Du Q. (2019). Discriminative Transfer Joint Matching for Domain Adaptation in Hyperspectral Image Classification. IEEE Geosci. Remote Sens. Lett..

[18] Peng J., Sun W., Du Q. (2019). Self-Paced Joint Sparse Representation for the Classification of Hyperspectral Images. IEEE Trans. Geosci. Remote Sens..

[19] Zhang S., Wu R., Xu K., Wang J., Sun W. (2019). R-CNN-Based Ship Detection from High Resolution Remote Sensing Imagery. Remote Sens..

[20] Mao Q., Hu F., Hao Q. (2018). Deep learning for intelligent wireless networks: A comprehensive survey. IEEE Commun. Surv. Tutorials.

[21] Zhang C., Patras P., Haddadi H. Deep Learning in Mobile and Wireless Networking: A Survey. https://ieeexplore.ieee.org/abstract/document/8666641.

[22] Goussevskaia O., Halldórsson M.M., Wattenhofer R., Welzl E. Capacity of arbitrary wireless networks. Proceedings of the IEEE International Conference on Computer.

[23] Xu X., Tang S., Wan P. Maximum weighted independent set of links under physical interference model. Proceedings of the International Conference on Wireless Algorithms, Systems, and Applications.

[24] Pei G., Vullikanti A. Low-complexity scheduling for wireless networks. Proceedings of the ACM International Symposium on Mobile Ad Hoc Networking and Computing.

[25] Joo C., Lin X., Ryu J., Shroff N.B. (2016). Distributed Greedy Approximation to Maximum Weighted Independent Set for Scheduling with Fading Channels. IEEE/ACM Trans. Netw..

[26] Wan P.J., Yuan H., Jia X., Wang J., Wang Z. Maximum-weighted subset of communication requests schedulable without spectral splitting. Proceedings of the IEEE Conference on Computer Communications.

[27] Deng H., Yu J., Yu D., Li G., Huang B. (2015). Heuristic Algorithms for One-Slot Link Scheduling in Wireless Sensor Networks under SINR. Int. J. Distrib. Sens. Netw..

[28] Chackochan R., Dhanasekaran S., Sunny A. (2018). Asynchronous Distributed Greedy Link Scheduling in Multihop Wireless Networks. IEEE Trans. Veh. Technol..

[29] Chen X.-W., Lin X. (2014). Big data deep learning: challenges and perspectives. IEEE Access.

[30] Kotenko I., Saenko I., Branitskiy A. (2018). Applying Big Data Processing and Machine Learning Methods for Mobile Internet of Things Security Monitoring. J. Internet Serv. Inf. Secur..

[31] Kumar N., Swain S.N., Murthy C.S.R. (2018). A Novel Distributed Q-Learning Based Resource Reservation Framework for Facilitating D2D Content Access Requests in LTE-A Networks. IEEE Trans. Netw. Sci. Eng.

[32] Liu L., Yin B., Zhang S., Cao X., Cheng Y. (2018). Deep Learning Meets Wireless Network Optimization: Identify Critical Links. IEEE Trans. Netw. Sci. Eng..

[33] Khan M., Alam M., Moullec Y., Yaacoub E. (2017). Throughput-Aware Cooperative Reinforcement Learning for Adaptive Resource Allocation in Device-to-Device Communication. Future Internet.

[34] Cui W., Shen K., Yu W. (2019). Spatial Deep Learning for Wireless Scheduling. IEEE J. Sel. Areas Commun..

[35] Ye H., Li G.Y. Deep Reinforcement Learning for Resource Allocation in V2V Communications. Proceedings of the IEEE International Conference on Communications (ICC).

[36] Belotti P., Bonami P., Fischetti M., Lodi A., Monaci M., Nogales-Gómez A., Salvagnin D. (2016). On handling indicator constraints in mixed integer programming. Comput. Optim. Appl..

[37] Shih W. (1979). A branch and bound method for the multiconstraint zero-one knapsack problem. J. Oper. Res. Soc..

[38] Toth P. (1980). Dynamic programming algorithms for the zero-one knapsack problem. Computing.

[39] Camm J.D., Raturi A.S., Tsubakitani S. (1990). Cutting big M down to size. Interfaces.

[40] Klotz E., Newman A.M. (2013). Practical guidelines for solving difficult mixed integer linear programs. Surv. Oper. Res. Manag. Sci..

[41] Date P., Hendler J.A., Carothers C.D. (2016). Design index for deep neural networks. Procedia Comput. Sci..

[42] Srivastava N., Hinton G., Krizhevsky A., Sutskever I., Salakhutdinov R. (2014). Dropout: A simple way to prevent neural networks from overfitting. J. Mach. Learn. Res..

[43] LeCun Y., Bengio Y., Hinton G. (2015). Deep learning. Nature.

[44] Hinton G.E., Osindero S., Teh Y.W. (2006). A fast learning algorithm for deep belief nets. Neural Comput..

